# Ultrasonographic examination of plantar fasciitis: a comparison of patient positions during examination

**DOI:** 10.1186/s13047-016-0171-4

**Published:** 2016-09-15

**Authors:** Jae Hoon Ahn, Choong Woo Lee, ChanJoo Park, Yoon-Chung Kim

**Affiliations:** 1 Department of Orthopaedic Surgery, Seoul St. Mary’s Hospital, College of Medicine, The Catholic University of Korea, 222, Banpo-daero, Seocho-gu, Seoul 06591 Republic of Korea; 2 Department of Orthopaedic Surgery, St. Vincent’s Hospital, College of Medicine, The Catholic University of Korea, Jungbu-daero 93, Paldal-gu, Suwon-si, Gyeonggi-do 16247 Republic of Korea

**Keywords:** Plantar fasciitis, Ultrasound, Supine position, Prone position

## Abstract

**Background:**

Musculoskeletal ultrasound is a non-invasive and low-cost modality for real-time visualisation of the plantar fascia. Ultrasound examination for plantar fasciitis is generally performed with the patient in a prone position, although the rational for using a prone position has not been validated. The aim of the study was to investigate if ultrasound examination in a supine position, which is more comfortable than the prone position, is valid.

**Methods:**

We conducted a prospective study of 30 participants with plantar fasciitis, 8 men (27 %) and 22 women (73 %), with a mean age of 53.9 ± 12.6 (range, 32 to 77) years, and an equal distribution of left and right feet. The plantar heel was divided into three portions for ultrasound examination: medial, central and lateral. Two measurements of plantar fascia thickness were obtained for each portion, with participants in 2 positions (supine and prone) and for 2 ankle postures (neutral and 15° of plantarflexion). Mean measurements of plantar fascia thickness were compared between the two positions (Wilcoxon signed rank tests for non-normally distributed data and paired *t*-tests for normally distributed data). Participants were asked to report their preferred position for examination, supine or prone.

**Results:**

The measured thickness was comparable for both supine and prone positions, for both ankle postures, neutral and 15° of plantarflexion (*p* > 0.05). A specific self-reported preferred position was not identified.

**Conclusions:**

Ultrasound examination of plantar fasciitis can be performed in the supine position without any significant difference in measurement compared to examination in the conventional prone position.

**Trial registration:**

The Catholic Medical Center Office of Human Research Protection Program (CMC-OHRP)/Institutional Review Board approved the current study (Approval No. KC12DISI0338), and all participants provided their written informed consent for participation and publication.

## Background

Plantar fasciitis is the most common cause of chronic heel pain, accounting for 15 % of all foot complaints among out-patients in general orthopaedic clinics [1–5]. Histologically, the plantar fascia is comprised of a common tendon aponeurosis for a superficial layer of intrinsic plantar foot muscles, rather than being a true fascial layer.

As an aponeurosis, the plantar fascia is easily visualised by ultrasound imaging, similar to superficial tendons. Musculoskeletal ultrasound is considered to be a useful diagnostic modality for plantar fasciitis, providing real-time and dynamic visualisation, while being non-invasive, inexpensive and radiation-free [1, 5, 6]. Generally, ultrasound examination of plantar fasciitis is performed with the patient in a prone position. However, no theoretical background or validation studies have been conducted to confirm the most appropriate position for ultrasound examination of plantar fasciitis. During ultrasound examination for plantar fasciitis, the authors have found that the prone position is relatively time-consuming because the patient has to change position after sitting on the table. In addition, the supine position appears to be more comfortable compared to the prone position since the chest and abdomen are not compressed as they are in the prone position.

The aim of the study was to investigate the concurrent validity (accuracy) of conducting an ultrasound examination of the plantar fascia in a supine position. If found to be valid, the supine position may be of benefit for reducing examination time and improving patient comfort during examination.

## Methods

Our methods and procedures were approved by the Catholic Medical Center Office of Human Research Protection Program (CMC-OHRP)/Institutional Review Board (Approval No. KC12DISI0338), and all participants provided written informed consent for participation and publication of the research findings.

The study sample was comprised of 30 participants who underwent ultrasound examination for plantar fasciitis in our clinic, between June 2012 and January 2013. Inclusion criteria were: (i) chronic heel pain > 3 months; (ii) presence of morning pain characteristic of plantar fasciitis; (iii) confirmation of heel pad tenderness on physical examination; and (iv) thickness of the plantar fascia > 4.0 mm on ultrasound imaging. This cut-off thickness criterion was based on current evidence that a plantar fascia thickness > 4.0 mm on ultrasound imaging is consistent with plantar fasciitis [1, 6–9]. Exclusion criteria were: systematic inflammatory arthritis, diabetes mellitus and long-standing neuromuscular disease. Our sample included 8 men (27 %) and 22 women (73 %), with a mean age of 53.9 ± 12.6 (range, 32 to 77) years, with an equal distribution of right and left presentation of plantar fasciitis (*n =* 15 each). Prior to ultrasound imaging at the first clinic visit, duration of symptoms and the Visual Analogue Scale pain scores were recorded. Following the ultrasound examination, participants were asked to state their preferred examination position, supine or prone.

The ultrasound examination was performed using a Philips HD11-XE Ultrasound System (Koninklijke Philips Electronics N.V., Amsterdam, the Netherlands), fitted with a 12-MHz linear-array transducer. For assessment, the plantar heel was divided into medial, central and lateral portions. Considering that the measured thickness of plantar fascia may be influenced by the ankle posture, measurements for each portion of the plantar heel were obtained in two ankle joint postures, neutral and 15° of plantarflexion. Each measurement was repeated twice, for the two ankle postures and for the two positions, with the mean of the two measurements used to compare measurements obtained in supine and in prone.

**Fig. 1:**
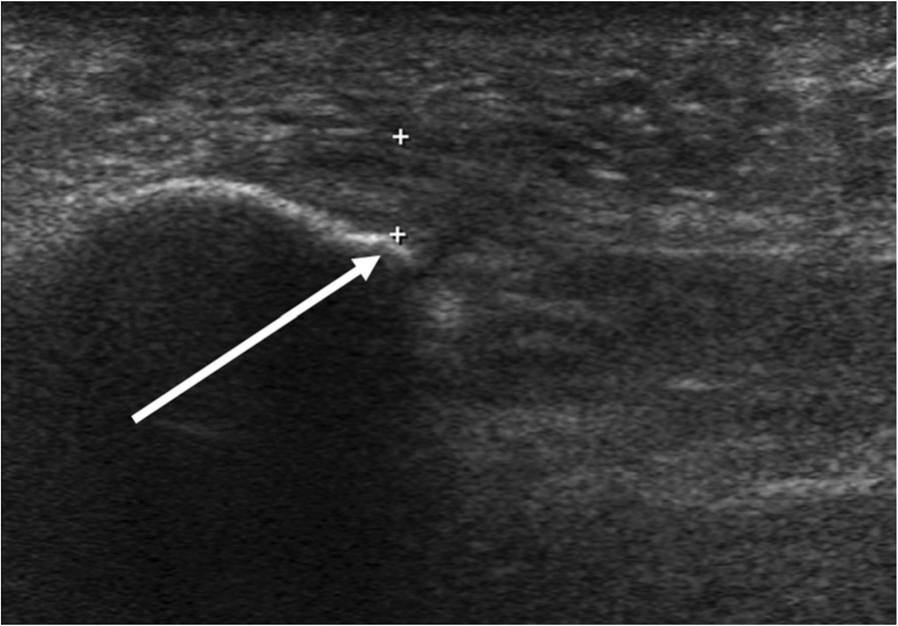
The vertical thickness of the plantar fascia was measured at a standard reference point (*white arrow*) where the fascia crosses the anterior most aspect of the inferior border of the calcaneus

All measurements of plantar fascia thickness were obtained at the same reference point, where the fascia crosses the anterior most aspect of the inferior border of the calcaneus [1]. The vertical thickness of the plantar fascia was measured as shown in Fig. 1, with confirmation that all participants had a thickness > 4.0 mm. All ultrasound examinations and measurements were performed by one sonographer. The sonographer (Y-CK) was a second-year fellow in a foot and ankle subspecialty and a certified medical doctor from the national board of orthopedic surgery. In addition, the sonographer had finished basic and advanced musculoskeletal ultrasound courses, which were hosted by the national academic society of foot and ankle ultrasound imaging. During our study, every measurement was double-checked by the senior author (JHA) who is an executive member of the national academic society of foot and ankle ultrasound imaging. The senior author has more than 10 years of experience in performing ultrasound examination of plantar fasciitis.

All data were analysed using SPSS version 18.0 (SPSS Inc. Chicago, Illinois). Paired *t*-tests were used for normally distributed data and Wilcoxon signed rank tests were used for non-normally distributed data to compare the supine and prone position groups. Means and standard deviations were computed for normally distributed variables, whereas medians and the interquartile range (IQR; 25th-75th) were used for non-normally distributed data. Statistical significance was accepted for *p* values of < 0.05.

## Results

The mean duration of symptoms prior to the clinic visit was 8.4 ± 9.07 months (range 2 to 48 months), with a mean Visual Analogue Scale pain score of 6.1 ± 1.67 (range 3 to 9). With regard to positioning for the ultrasound examination, 14 participants (47 %) preferred the supine position and 13 (43 %) the prone position, with the remaining 3 participants (10 %) not having a preference. Participants provided the following reasons for preferring the supine position for the examination: (i) it facilitated communication with the examiner during the ultrasound examination and (ii) it provided the possibility of seeing abnormal findings on the monitor; combined with real-time explanation regarding the status of their painful heel.

**Fig. 2:**
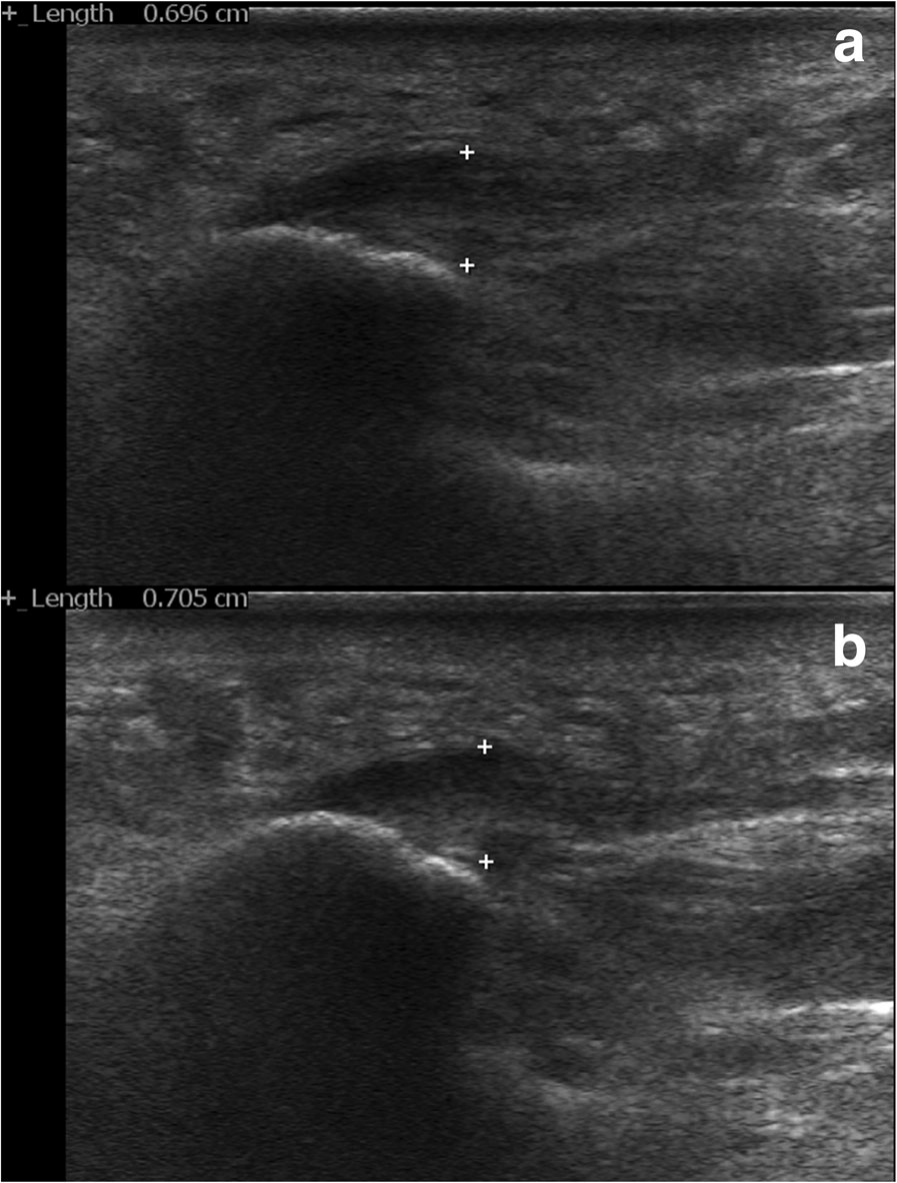
Comparison of measurements of plantar fascia thickness obtained in supine and in prone positions for a representative 54-year-old woman, with the ankle in a neutral posture. A thickness of 7.0 mm was obtained in the supine position (**a**); compared to 7.1 mm in the prone position (**b**)

The measured thickness of the plantar fascia in both supine and prone positions, with the ankle in a neutral posture, is shown in Fig. 2, with no between-position differences in measurements across all three portions of the plantar heel (*p* > 0.05; Table 1). Comparable results were also obtained for measurements between supine and prone positions, with the ankle posture of 15° plantarflexion (*p* > 0.05; Table 2).

**Table 1:** Comparison of plantar fascia thickness between supine and prone positions with the ankle in a neutral posture

Portion of plantar heel	Position		*p* value
	Supine	Prone	
Medial	5.0 [4.5; 6.0]	5.1 [4.2; 6.1]	0.973^a^
Central	5.1 [4.1; 5.6]	5.0 [4.2; 6.1]	0.620^a^
Lateral	4.9 ± 1.0	4.8 ± 1.1	0.600^b^

Measured thickness (mm) values are presented as median [interquartile range; 25th–75th] for non-normally distributed data or mean ± standard deviation for normally distributed data

*p* values obtained from Wilcoxon signed rank test ^a^ or paired *t*-test ^b^

Significance was accepted for *p* values of < 0.05

**Table 2:** Comparison of plantar fascia thickness between supine and prone positions with the ankle in a 15° plantarflexion posture

Portion of plantar heel	Position		*p* value^§^
	Supine	Prone	
Medial	5.0 [4.3; 5.8]	5.0 [4.4; 6.0]	0.052
Central	4.7 [4.2; 5.4]	4.9 [4.2; 5.4]	0.062
Lateral	4.5 [4.1; 5.0]	4.6 [4.1; 5.1]	0.674

Measured thickness (mm) values are presented as median [interquartile range; 25th–75th] as data was not normally distributed

^§^ *p* values were obtained from Wilcoxon signed rank test

Significance was accepted for *p* values of < 0.05

## Discussion

The plantar fascia is easily visualised by ultrasound imaging because it is a superficial structure. Sabir et al. [1] suggested that ultrasound imaging could be as valuable as magnetic resonance image for the diagnosis of plantar fasciitis. As the ultrasound examination is readily available, it is increasingly being used for the diagnosis of plantar fasciitis [6, 9].

The usefulness of ultrasound imaging is often limited by examiner-dependent error [10]. However, previous studies have provided evidence of the reproducibility of measurements of plantar fascia thickness by ultrasound, with high intra-and inter-observer reliability [11, 12]. In our study, all ultrasound measurements were performed by one examiner to eliminate examiner-dependent variability in measurement.

In the majority of studies reported to date, ultrasound examination of plantar fasciitis has been performed with the patient in a prone position, with the foot hanging freely over the end of the examination bed at an angle of 90° relative to the leg [1, 5, 6, 9, 13]. However, evidence to support this position is lacking, which prompted us to undertake our investigation. In this comparative study, we investigated whether there were any significant differences in ultrasound measurements of plantar fascia thickness when participants were placed in a supine or a prone position.

Our findings provide evidence that measurements obtained in both the supine and prone position were similar, with differences in measurement of 0.1 mm in the medial, central and lateral portions of the plantar fascia with the ankle in a neutral posture, and no more than 0.2 mm for the central portion and 0.1 mm for the lateral portion with the ankle posture of 15° plantarflexion. Moreover, a preferred position was not definitively identified by participants, although participants did report viewing of the ultrasound images as a benefit of examination in the supine position. However, some participants reported feeling ‘awkward’ and ‘nervous’ facing the examiner when the examination was performed in the supine position.

Based on these results, we propose that the either a supine or prone position can effectively be used for the ultrasound examination of plantar fasciitis, with the position not being a significant influence on the accuracy of measurements. Therefore, the position for ultrasound examination can be selected in a patient-specific way without concern for the accuracy of the measurements. In particular, if the patient is unable to or if they feel uncomfortable being requested to adopt the prone position, the ultrasound examination of plantar fasciitis can be performed in the supine position knowing that the result will be the same.

There are three limitations of this study that should be acknowledged. Firstly, our study sample was relatively small (*n =* 30), with a predominance of women (73 %). Secondly, only two measurements of plantar fascia thickness were obtained for each portion of the plantar heel, with measurements obtained during the same assessment session. Therefore, intra-observer reliability was not formally addressed. Thirdly, the sonographer could not perform all measurements in a blinded manner because the out-patient clinic examination was performed simultaneously with the ultrasound examination. With these limitations in mind, we consider our study as providing preliminary evidence and justification for larger studies, with a blinded assessor (or preferably blinded assessors), to confirm the equivalence of the two patient positions, supine and prone, for ultrasound examination of plantar fasciitis.

## Conclusions

We found no difference in the ultrasound measurement of plantar fascia thickness in our sample of participants with plantar fasciitis when the examination was performed in the prone or the supine position. In addition, participants did not have a clear preference for whether they preferred to be examined in the prone or supine positions. Therefore, ultrasound examination of plantar fasciitis can be performed with patients in either a supine or a prone position, according to patients’ preference, without concern about the accuracy of the measurement.

## Abbreviations

CMC-OHRP: The catholic medical center office of human research protection program
